# Antibiotic treatment at delivery shapes the initial oral microbiome in neonates

**DOI:** 10.1038/srep43481

**Published:** 2017-02-27

**Authors:** Luisa F. Gomez-Arango, Helen L. Barrett, H. David. McIntyre, Leonie K. Callaway, Mark Morrison, Marloes Dekker Nitert

**Affiliations:** 1 School of Medicine, The University of Queensland, Brisbane Australia; 2 UQ Centre for Clinical Research, The University of Queensland, Brisbane Australia; 3 Obstetric Medicine, Royal Brisbane and Women’s Hospital, Brisbane Australia; 4 Mater Research, The University of Queensland, Brisbane Australia; 5 Diamantina Institute, Faculty of Medicine and Biomedical Sciences, The University of Queensland, Brisbane Australia; 6 School of Chemistry and Molecular Biosciences, The University of Queensland, Brisbane Australia

## Abstract

Oral microorganisms are important determinants of health and disease. The source of the initial neonatal microbiome and the factors dictating initial human oral microbiota development are unknown. This study aimed to investigate this in placental, oral and gut microbiome profiles from 36 overweight or obese mother-baby dyads as determined by 16S rRNA sequencing. Expression of five antibiotic resistance genes of the β-lactamase class was analysed in the infant oral microbiota samples by QPCR. The neonatal oral microbiota was 65.35% of maternal oral, 3.09% of placental, 31.56% of unknown and 0% of maternal gut origin. Two distinct neonatal oral microbiota profiles were observed: one strongly resembling the maternal oral microbiota and one with less similarity. Maternal exposure to intrapartum antibiotics explained the segregation of the profiles. Families belonging to Proteobacteria were abundant after antibiotics exposure while the families *Streptococcaceae, Gemellaceae* and *Lactobacillales* dominated in unexposed neonates. 26% of exposed neonates expressed the *Vim-1* antibiotic resistance gene. These findings indicate that maternal intrapartum antibiotic treatment is a key regulator of the initial neonatal oral microbiome.

Evidence of intrauterine microbial colonization has challenged the paradigm that fetal development occurs in a sterile environment. Mothers are the primary source of bacteria for newborns but it is unclear whether the structuring of the infant microbiome is the result of mother-to-newborn transmission prior, during or after birth^1^. The presence of bacterial communities in meconium^2^,^3^,^4^, amniotic fluid^5^, placenta^6^,^7^,^8^, fetal membranes^9^ and the fact that the maternal microbiota is needed to shape offspring’s immune system^10^, all point to a host-microbial interaction *in utero*. Disruption of microbiota transmission from mother to infant has been associated with obesity^11^,^12^,^13^, type I diabetes^14^,^15^ asthma^16^,^17^ and neurodevelopment^18^ later in life.

Identifying pioneer colonizers is essential to elucidate the early stages of microbiota development. The initial inoculation comprises both beneficial and pathogenic bacteria and may condition subsequent colonization thereby shaping the microbiome for adulthood^19^. While the colonization process of the infant gut microbiome has been studied extensively, the colonization of the neonatal oral microbiome is still unclear. Optimal infant intestinal establishment begins with oral inoculation by maternal microbiota^20^, highlighting the importance of the oral-systemic connection in infants.

The first microbial colonizers of the oral cavity in the first day of life stimulate changes in the oral cavity that favour the growth of subsequent species^21^ with species from the genus *Streptococcus* predominating the initial oral microbial load^22^,^23^,^24^. These pioneer species usually bind to mucosal epithelium (e.g. *Streptococcus salivarius*) where they produce extracellular polymers from sucrose to which other bacteria can attach^25^. This causes a selective pressure for certain bacteria in the oral cavity. The acquisition of the first microbial colonizers may be altered by multiple maternal and infant factors, which may result in differences in oral microbiota development. These factors could include mode of delivery, breastfeeding, pregnancy outcomes, parental contact, antibiotics, host factors and living environment, all of which have been recognised to affect infant’s intestinal microbial habitat^19^,^26^,^27^,^28^,^29^. It is however not clear if, and to what extent, these factors contribute to shaping the oral microbiome.

In the present study, plausible sources for the seeding of the neonatal oral microbiome in the first days of life were investigated through profiling the placental, maternal gut and oral microbiome profiles from mother-baby dyads from the SPRING cohort^30^. In addition, maternal, pregnancy and infant factors that may shape the infant oral microbiota were analysed.

## Results

Maternal and infant clinical characteristics from the 36 healthy mother-infant pairs included in this study are shown in Table 1. All women were of Caucasian ethnicity and were overweight (41.7%) or obese (58.3%). Almost all of the women (30 of 36) exceeded gestational weight gain recommendations of the Institute of Medicine^31^ but none were diagnosed with gestational diabetes mellitus at 28 weeks gestation. Intrapartum antibiotics were administered to 63.9% of all women, with cephazolin (n = 15) and benzylpenicillin (n = 6) the most commonly prescribed. Of the 36 newborns included in this study, 55.6% were born by vaginal delivery, 44.4% by elective caesarean section and all were born at term. With the exception of one infant, all infants were breastfed. Birth weight was within normal range and 65% of newborns were male.

**Figure 1:**
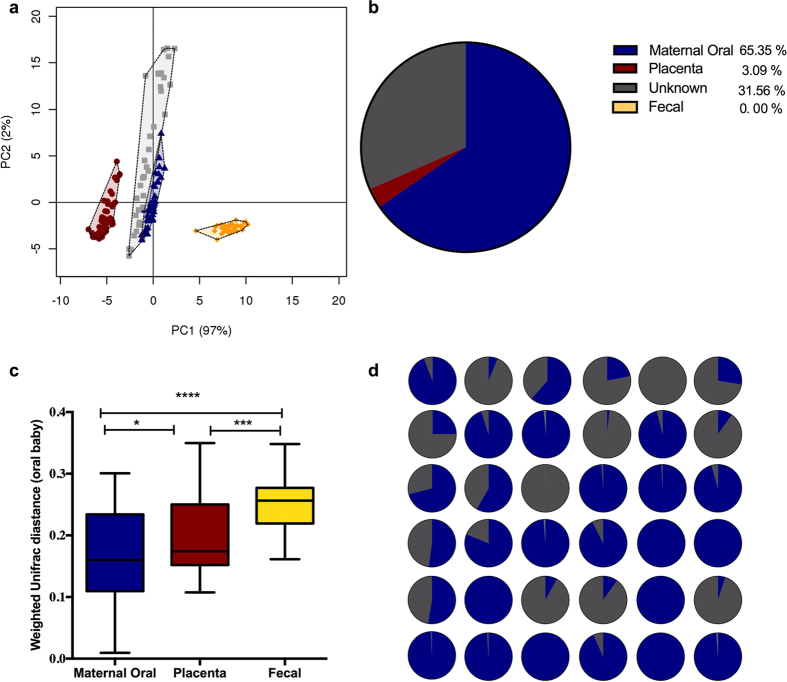
Maternal and neonatal microbial community analysis. (**a**) Principal component analysis (PCA) plot for maternal feces (yellow), maternal oral (blue), placenta (red) and infant oral (pale grey). Significant differences were reported between groups of samples (R = 0.88, *p* = 0.001). (**b**) Proportion of maternal oral (blue), placenta (red), maternal feces (yellow) and unknown source of environment (grey) in neonates oral microbiota using bacterial source tracking. (**c**) Bacterial community distances (weighted Unifrac distances) between maternal feces (yellow), placenta (red) and maternal oral (blue) with respect to infant’s oral microbiota. Boxplots shows the 25^th^ and 75^th^ percentile with a line at the median. (**d**) Proportion of maternal oral microbiota (blue) and unknown source of environment (grey) in each neonates oral microbiota using bacterial source tracking.

In total 36 mother-infant pairs were selected from the SPRING cohort for having a complete matched sample set of placenta, maternal oral swab and infant oral swabs with 16 pairs also having maternal fecal samples available. PCA and identification of clustering was performed for all four microbiomes together (Fig. 1a). Four distinct niches (representing gut, placenta, maternal oral and infant oral microbiota) were present in the PCA analysis, demonstrating the critical role of the varying physicochemical characteristics of the different sites in defining bacterial composition (R = 0.88, *p* = 0.001). When comparing the bacterial community distances between the niches, there was less distance between the bacterial communities of the maternal and neonatal oral microbiota compared to the placental and gut microbiota (*p* = 0.0001) (Fig. 1b). Bacterial source tracking analyses were also used to examine the similarity of the maternal oral and gut microbiota, as well as the placental microbiota, to the infant’s oral microbiota. These analyses showed that the neonatal oral microbiota profile is comprised principally of maternal oral microbiota (65.35%), and OTUs of “unknown” (environmental) origin (31.56%). Neither placental (3.09%) nor maternal gut OTUs (0%) were prominent in the neonate oral microbiota (Fig. 1c). Despite the obvious influence of the mother’s oral microbiota on most of the neonate profiles, we still observed diversity across the cohort with respect to the degree of overlap between a mother’s and infant’s oral microbiota profiles. The cohort was divided into two groups based on similarity of the infant oral microbiota to the maternal oral microbiota: the high-similarity group which had a similarity above the median similarity of 92.8% (median 98.8; IQR 95.6–00%, n = 19) and the low similarity group which had a similarity below the median of 92.8% (median 22.0; IQR 5.7–55.4%, n = 17; Fig. 1d and Supplementary Table 1).

**Figure 2:**
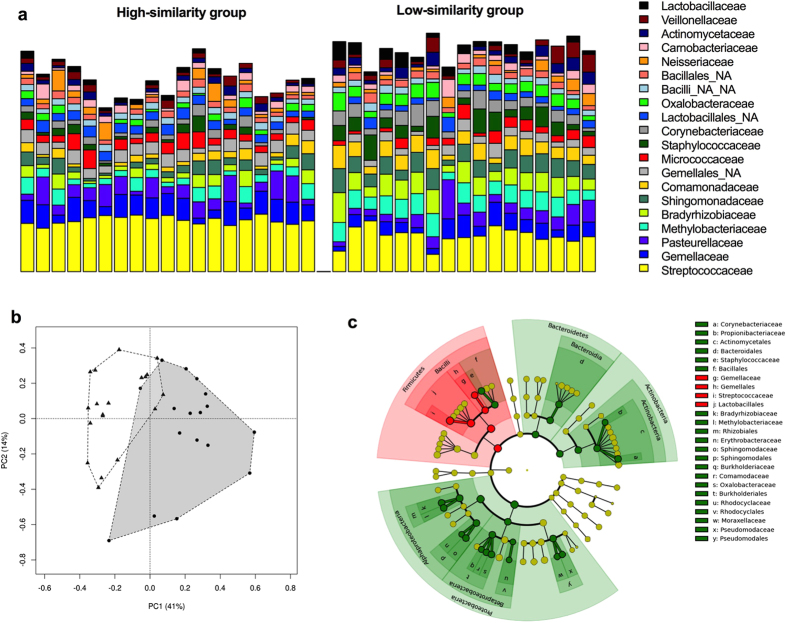
Newborn’s oral microbiome composition, structure and differences in abundance of bacterial communities at family level. (**a**) Relative abundances of bacterial families in the newborn’s oral microbiota. Infants oral microbiota was subdivided into 2 groups based on high similarity in taxonomy (group 1, n = 19) or less similarity (group 2, n = 17) to their corresponding maternal oral microbiota. (**b**) Principal component analysis (PCA) plot at family level for group 1 and group 2. Significant differences were reported between both groups (R = 0.52, *p* = 0.001). (**c**) Cladogram generated by LEfSe indicating differences in taxa between group 1 and group 2. Each successive circle represents a phylogenetic level (phylum, class, order, family, genus). Regions in red indicate taxa enriched in group 1 while regions in green indicate taxa enriched in group 2. Different taxa (at family and order level) are listed on the right side of the cladogram.

Although taxa assigned to the *Firmicutes* and *Proteobacteria* phyla were the most prevalent members of the neonatal oral microbiota (Supplementary Figure 3) in both similarity groups, they could be clearly differentiated from each other. First, the alpha diversity metrics produced from the profiles assigned to the low-similarity group indicate there is a greater richness (*p* = 0.003) and diversity (*p* < 0.0001) (Supplementary Figure 4) within these samples, compared to the mother-baby dyads assigned to the high-similarity group. The profiles of the high-similarity group were distinguished by an enrichment of taxa assigned to the phyla *Firmicutes (p* < 0.0001) and *Actinobacteria (p* < 0.001), whereas taxa assigned to the phyla *Bacteroidetes (p* < 0.01) and *Proteobacteria (p* < 0.0001) were significantly enriched in the lower-similarity group (Supplementary Figure 3). A total of 16 different family level phylotypes were detected in the neonatal oral microbiota (R = 0.52, *p* = 0.001, Fig. 2a,b; Supplementary Figure 5). The high-similarity group was significantly enriched with the families *Gemellaceae* and *Streptococcaceae* and the order *Lactobacillales*. The low-similarity group exhibited higher abundances of 10 families belonging to phylum Proteobacteria, as well as a greater abundance of OTUs assigned to the families *Corynebacteriaceae* and *Propionibacteriaceae*, and *Staphylococcaceae* (Fig. 2c and Supplementary Figure 5).

**Figure 3:**
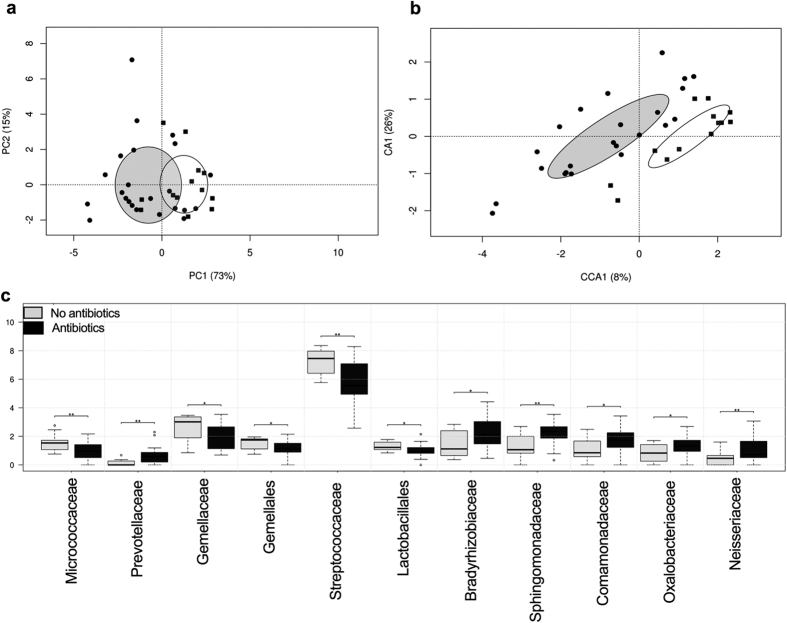
Maternal antibiotic exposure at delivery drives the differences in neonatal oral microbiota. (**a**) Principal component analysis (PCA) plot for infants exposed to maternal antibiotics (•) and non-exposed infants (▪). Significant differences were reported between both groups (R = 0.21, *p* = 0.02). (**b**) PCA plot indicates that samples cluster by exposure or non to intrapartum antimicrobials. The statistical significance of the observed clustering was further confirmed by canonical correspondence analysis (CCA) (*p* = 0.03). (**c**) Significant different taxa are shown as boxplots (25^th^ and 75^th^ percentile with a line in the median). Pair-wise comparison were done by t-test and annotated as *p < 0.05, **p < 0.01.

Numerous maternal and infant factors were used as constraints for the microbiota data, in order to investigate their possible contribution to the segregation of the neonatal oral microbiomes. Of these measures, administration of intrapartum antibiotics showed a significant difference between groups (*p* = 0.04), and was higher among infants assigned to the low-similarity group (Table 2). In total, 23 mothers were treated with antibiotics in the peripartum period: 16/16 delivering by C-section and 7/20 delivering vaginally. The medical reasons why women who delivered vaginally received antibiotics intrapartum include prophylactic use according to hospital protocol, prolonged rupture of membranes, and infections. These differences were also clearly revealed by PCA analysis (Fig. 3a). Adonis testing further supported the clustering of the neonatal oral microbiota on the basis of maternal exposure to antibiotics at delivery (R = 0.21, *p* = 0.02) but not with respect to mode of delivery (Table 2, Supplementary Figure 6, R = 0.03, p = 0.70). The statistical significance of this clustering was further confirmed by canonical correspondence analysis (*p* = 0.03, Fig. 3b), and the significant differences in the relative abundances of the bacterial taxa reported above were also consistent with respect to a mother’s intrapartum antibiotic use. More specifically, the families *Streptococcaceae, Gemellaceae* and order *Lactobacillales* were enriched in infants whose mothers were not exposed to antibiotics, whereas families from the phylum *Proteobacteria* were significantly more abundant in infants whose mothers used antibiotics (Fig. 3c). The different classes of antibiotics administered did not affect the bacterial community structure in the neonatal oral microbiota (R = 0.039, *p* = 0.62) (Supplementary Figure 7). However, the oral microbiomes of the infants of two mothers who were given a cocktail of antibiotics (Cephazolin, Benzylpenicillin and Metronidazole) clustered separately from the infants whose mothers received only one antibiotic.

The oral microbiome DNA of all the neonates were examined for the presence of 5 different beta-lactamase genes by PCR: *Vim-1, Cmy-2, Oxa-1, Shv* and *Tem.* Expression of *Vim*-1 was detected in 6/36 newborns (Table 2). These results were confirmed using QuantiTect Probe PCR as described in the methods. Notably, the mothers of all six neonates expressing *Vim-1* in the oral microbiomes had received antibiotics intrapartum, Three were treated with Benzylpenicillin with Benzylpenicillin, and three with Cephazolin.

## Discussion

In the present study, we use cultivation-independent techniques to characterise the initial microbial oral community in term neonates and compare it with their mothers’ microbiota from different body sites. Only limited information is available on the oral microbiome development in neonates. This study demonstrates that the infant’s oral cavity is rapidly colonized by bacteria resembling those in their mother’s oral cavity in a cohort of overweight and obese women. This may indicate that the first bacteria in infant’s mouth are of maternal origin, or that the oral cavity microenvironment specifically stimulates colonization by specific bacteria. The interindividual microbial variability was high among infants; however, maternal intrapartum antibiotic administration significantly contributed to the shaping of the microbial colonization pattern in the neonatal oral cavity.

Broad-spectrum antibiotics are commonly used prophylactically during intrapartum care to reduce the risk for infectious disease^32^. Currently, more than 40% of pregnant women receive antibiotics in the intrapartum period^33^; however, the immediate and long-term effects of antibiotic treatment during pregnancy and at delivery on either the maternal and infant microbiome have not been studied extensively^34^. Emerging evidence shows that antibiotic administration to mothers during labour significantly affects the development of the intestinal microbiota in preterm neonates^35^, reduces intestinal host defences^1^ and leads to alterations in the vaginal microbiota prior to birth^36^. To our knowledge, this is the first study to confirm that the effects of intrapartum antibiotics also influence the oral microbiota composition in neonates irrespective of the mode of delivery. Holgerson *et al*.^37^ reported that differences in baby’s oral microbiota were associated with mode of delivery; however, since all women delivering by C section received antibiotics in that study vs. none of those who delivered vaginally it is possible that the results reflect antibiotic use rather than mode of delivery. Since antibiotics are routinely administered to mothers delivering by caesarean section to reduce the risk of peripartum infections^32^, this constitutes a confounder in other studies reporting delivery mode as the main driving factor for differences in infant microbiome development.

The two main antibiotics administered intrapartum in this study are Cephazolin and Benzylpenicillin. These antibiotics are among the most commonly prescribed antimicrobials in Australia^38^. Their spectrum of activity includes both Gram-negative and Gram-positive microorganisms targeting diverse strains of Streptococci. This could explain the decreased abundance of the family *Streptococcaceae* and the greater diversity in the oral microbiota of infants from mothers who were given intrapartum antibiotics. As a result in the absence of the *Streptococcaceae* family, families belonging to phylum Proteobacteria colonize the infant’s oral cavity. This pattern is often regarded as a signature of dysbiosis and inflammation^39^. The families *Bradyrhizobiaceae, Sphingomonadaceae, Comamonadaceae, Neisseriaceae* and *Oxalobacteriaceae* are commonly isolated from hospitals^40^,^41^,^42^, and harbor possible opportunistic human pathogens. In contrast, the families *Streptococcaceae, Gemellaceae* and order *Lactobacillales* were all significantly enriched in neonates whose mothers were not exposed to antibiotics. Of note, oral Streptococci are widely recognized as one of the key group of early colonizers^43^, and their adhesion to surfaces within the oral cavity promotes the establishment of later colonizers, by their secretion of polysaccharides and adhesins that recruit both Gram-positive and Gram-negative bacteria^44^. This allows for the formation of a mature and stable biofilm community in the very early stages of human life. *Streptococcus* spp. can also metabolize milk carbohydrates^45^. Furthermore, maternal intrapartum antibiotic use has been reported to decrease the vertical transmission of the genus *Lactobacillus* to neonates^46^ and given the links between *Lactobacillus* establishment in neonates and infants with combatting allergy development later in life^47^ our findings reveal the need to consider how best to ensure restoration of these microbes in neonates. Lastly, the family *Gemellaceae* is predominantly abundant in the buccal epithelium of adults^48^ but little is known about their role in newborns. These results indicate that the use of narrow-spectrum antibiotics may be advisable in order to limit unintentional effects on the development of the initial microbiota in the neonate.

A critical outcome of antibiotic usage is the spread of antibiotic resistance genes, which is a serious public health concern. Here, neonatal oral microbiota DNA was screened for the presence of a limited group of genes encoding β-lactamases. The main antibiotics used for intrapartum treatment in this study belong to β-lactam group. Maternal antibiotic exposure was associated with expression of carbapenem-hydrolyzing enzymes of the *Vim*-1 gene in 6 of 23 neonates that received antibiotics. Previous studies have also reported the presence of *Vim*-type genes in infants^49^,^50^. Carbapenem-resistant isolates of *Pseudomonas aeruginosa* and other gram-negative non-fermenters have been identified to be *Vim*-1-containing microorganisms^51^. Further, the use of antibiotics in the first days after birth has been associated with high prevalence of late onset sepsis by potentially antibiotic resistant microorganisms such as *Pseudomonas*^52^. The presence of the family *Pseudomodaceae* in the low-similarity group could be the source of expression of the *Vim*-1 gene in this study. Regardless of the origin of *Vim*, this study provides evidence that the oral microbiota can harbor antibiotic resistance genes within days after delivery, which could result in microbial community instability later in life. From this study, it is however not possible to determine if the observed alterations in the oral microbiome profiles by intrapartum antibiotics in infants will last through childhood and disturb gut development. The effects of antimicrobials on bacterial succession, diversity and resistance are nevertheless known to endure long past infancy^53^, even after only a single dose^54^,^55^.

To our knowledge this is the first study assessing the impact of maternal obesity in neonatal oral microbiota. No associations with maternal BMI or gestational weight gain were found despite the fact that these factors^11^,^27^ have been associated with alterations in infants’ gut microbiota. Moreover, microbial sequences were identified in all placental tissues, suggesting that bacteria are transported to this fetal compartment and possibly provide the initial inoculum for the newborn’s microbiome development. Yet, only 3% of the bacteria in the infant’s oral cavity is of placental origin.

This study reveals that intrapartum antimicrobials modulate microbial colonization in newborns oral microbiome. Follow-up studies on the health of antibiotic-exposed infants should be considered for understanding the long-lasting effects on microbiota development and the spread of antibiotic resistance genes in newborns.

## Methods

### Study design and sample collection

This study is based on placental, oral and gut microbiome samples from 36 mother-baby dyads collected from overweight and obese pregnant women participating in the SPRING (the Study of Probiotics IN the prevention of Gestational diabetes) cohort (ANZCTR 12611001208998)^30^. Excess gestational weight gain was calculated for each woman as previously described^7^. Detailed demographic and clinical metadata were collected between baseline (<16 weeks gestation) and delivery. Refrigerated stool samples (n = 16) at 16 weeks gestation were self-collected by each woman and stored at −80 °C within one day after collection until DNA extraction. Maternal oral buccal swabs (n = 36) were taken at 36 weeks gestation and neonatal oral buccal swabs (n = 36) within 3 days after delivery. Each oral sample was collected by a sterile dry swab (Copan Diagnostics, Murrieta, CA), stored in a collection tube and placed immediately at −20 °C before transfer within 48 hours to −80 °C. Following delivery, term placentas (n = 36) were collected in sterile containers by trained midwifes under strict antiseptic protocol. Placentas were directly transferred to the laboratory. Trained researchers followed stringent sterile measures throughout the excision of placental samples. Placental membranes were removed and discarded to avoid contamination. Cuboidal 1 cm^3^ sections from the placental fetal side were excised and collected in autoclaved 1.5 mL tubes. Tubes were immediately placed in liquid nitrogen and stored at −80 °C until DNA extraction.

### Ethics, consent and permission

This study is approved by the Human Research Ethics committees of the Royal Brisbane and Women’s Hospital (HREC/RBWH/11/467) and The University of Queensland (2012000080). Written informed consent was obtained from all participants prior to enrolment in the trial. All experiments were performed in accordance with relevant guidelines and regulations.

### DNA extraction and processing

Genomic DNA was isolated from maternal feces, placental tissue and maternal and infant buccal swabs. Fecal microbial DNA from 0.25 grams of thawed stool sample was extracted by repeated bead beating and column (RBB + C) method followed by Qiagen AllPrep DNA extraction kit as detailed in ref. 56. DNA was isolated from buccal swabs and 100 grams of placental tissue by placing tissue in a 2 mL screw-cap tube containing 0.4 g of sterile zirconia beads (0.1 and 0.5 mm diameter) and 300 μL lysis buffer (NaCl 0.5 mol/L, Tris–HCl 50 mmol/L, pH 8.0, EDTA 50 mmol/L and SDS 4% w/v). Mechanical disruption was achieved by homogenization (Precellys, Bertin Technologies, Toulouse, France) for 3 min followed by incubation at 70 °C for 15 min. Oral and placental lysates were collected and transferred to the Maxwell 16 Buccal Swab LEV DNA Purification kit and to the Maxwell 16 Tissue DNA Purification kit (Promega, Madison, WI, USA) respectively, following the manufacturer’s recommendations. DNA extraction was carried out using the automated Maxwell 16 system. Contamination was monitored through assessment of reagent controls without addition of tissue or DNA. Extracted DNA was quantified by Nanodrop ND 1000 spectrophotometer (Nanodrop Technologies).

### 16S library preparation and sequencing

Fecal, oral, placental and control samples were assessed by high-throughput amplicon sequencing with dual index barcoding using Illumina MiSeq platform at the University of Queensland’s Australian Centre for Ecogenomics (ACE). The variable V6-V8 region was amplified using the primers 926F (5′-TCG TCG GCA GCG TCA GAT GTG TAT AAG AGA CAG AAA CTY AAA KGA ATT GRC GG-3′) and 1392R (5′-GTC TCG TGG GCT CGG AGA TGT GTA TAA GAG ACA GAC GGG CGG TGW GTR C-3′) yielding a product of 500 bps. Positive (*Escherichia coli* JM109) and negative (sterile deionized water) controls were included in each PCR run. Amplicons were cleaned using AMPure XP beads. PCR product were barcoded using Nextera XT v2 Index kit Set A and Set B. Human genomic DNA contamination was reduced by an additional purification step using Promega Wizard Gel Extraction Kit for placental and oral samples, followed by a second purification using AMPure XP beads. Libraries were quantified, normalized and pooled in equimolar amounts according to the manufacturer’s recommendations.

Bacterial sequences were analysed using QIIME (Quantitative Insights Into Microbial Ecology, v 1.9.1) pipeline^57^. Forward and reverse sequences for each sample were demultiplexed, quality filtered and joined. Operational taxonomic units (OTUs) were generated by open-reference OTU picking with 97% similarity using the Greengenes 13_8 database^58^. FastTree was used to construct phylogenetic trees. OTUs with overall relative abundance below 0.1% were removed from the OTU table. Reagent controls and PCR negative controls were included in the analyses. Detectable OTUs from negative controls were removed from each sample, generating a contamination free OTU table. Total numbers of sequence reads retained for analysis were 3,049,536 for maternal oral samples, 3,344,162 for infant oral, 39379 for placental samples and 1,004,548 for maternal feces. Cumulative sum scaling (CSS) normalization was applied to all samples as a differential abundance analysis^59^. Normalized OTU tables were then used for downstream analysis. Summaries of the taxonomic classification at five levels (Phylum, Class, Order, Family and Genus) were generated using default settings in QIIME. The sequences were deposited in the NCBI database (Accession number: Bioproject PRJNA357524).

### Bacterial source tracking

To investigate the contribution of maternal oral, placental and gut microbiota to the establishment of the neonatal oral microbiota, a Bayesian approach for bacterial source tracking (SourceTracker v 1.0)^60^ was used. Oral samples from neonates were designated as sinks and maternal samples (placental, oral and gut) of the corresponding mother were selected as sources.

### Screening for antibiotic resistance genes

Presence of five main antibiotic resistance genes (*Vim-1, Cmy-2, Oxa-1, Shv* and *Tem*) conferring β–lactamase resistance was assessed by real-time PCR in neonatal oral microbiota DNA samples. Primer sequences and annealing temperatures are listed in the Supplementary Table 2. Gene detection and amplification was carried out using 10 μL of SYBR Green PCR 2X master mix (Bio-Rad Laboratories), 1 μL of each primer (10 μM) and 20 ng of extracted oral DNA. DNA amplification was carried out using the following conditions: 1 cycle of 95 °C for 10 min, 40 cycles of denaturation at 95 °C for 10 s, annealing for 10 s and elongation at 72 °C for 10 s. Each reaction was run in duplicate. Positive controls and negative controls were added to each PCR run. QuantiTect Probe PCR, further validated presence or absence of *Vim*-1 gene on the Rotorgene Q real-time PCR instrument. The reaction mixtures consisted of 10 μL of QuantiTect Probe PCR Master Mix (Qiagen, Australia), 10 μM each forward and reverse primers, 0.2 μM of *Vim* probe 20 ng of extracted oral DNA (Table S1). Amplification and detection were achieved using the following two-step cycling conditions: an initial hold at 95 °C for 15 minutes, followed by 55 cycles at 95 °C for 15 s and 60 °C for 60 s.

### Statistical analysis

Microbiota diversity metrics were determined from normalised OTU tables. Alpha diversity was determined by the Chao 1 index for richness and Shannon-Wiener index for diversity. Microbial beta diversity was visualised by principal component analysis (PCA) using the Calypso software tool (http://bioinfo.qimr.edu.au/calypso/). The Adonis statistic for permutational multivariate analysis of variance available through Calypso was used to measure differences in variance between groups based on weighted UniFrac distances. The R-value measures the strength of the statistic test where 1 signifies total difference between groups. Statistical significance of any observed clustering was further tested by canonical correspondence analysis (CCA). Bar plots and boxplots at phylum and family level were generated to visualise the taxonomic distribution of the neonatal oral microbiome. The LDA Effect Size (LEfSe: Linear Discriminant Analysis Effect Size) algorithm was used to identify taxa with differentiating relative abundance using the online interface Galaxy (http://huttenhower.sph.harvard.edu)^61^. The threshold for the logarithmic LDA score was set at 3.0 for biomarker discovery. Analyses for clinical characteristics were performed with nonparametric testing and expressed as median with interquartile ranges (Q1–Q3). Comparison between categories was performed by Mann-Whitney U test and Fisher’s exact test to compare proportions. A *p* value < 0.05 was considered significant.

## Additional Information

**How to cite this article:** Gomez-Arango, L. F. *et al*. Antibiotic treatment at delivery shapes the initial oral microbiome in neonates. *Sci. Rep.* **7**, 43481; doi: 10.1038/srep43481 (2017).

**Publisher's note:** Springer Nature remains neutral with regard to jurisdictional claims in published maps and institutional affiliations.

## Supplementary Material

Supplementary Information

## Figures and Tables

**Table 1:** Maternal and infant clinical characteristics.

**Mother (n** = **36)**	
Age (years)	35.0 (33.0–37.0)
BMI (kg/m^2^)	30.8 (27.5–33.1)
Gestational age at delivery (wks)	39.7 (38.5–40.6)
Mode of delivery	
Vaginal	55.6%
Caesarean	44.4%
Intrapartum antibiotic	
Cephazolin	15/36 (41.7%)
Benzylpenicillin	6/36 (16.7%)
Others^a^	2/36 (5.5%)
None	13/36 (36.1%)
Gestational weight gain (kg)	8.7 (6.9–13.8)
**Infant (n** = **36)**	
Birth weight (g)	3685 (3306–3980)
Body Fat (%)	12.4 (9.6–16.1)
Gender	
Female	34.3%
Male	65.7%

Clinical characteristics of mother-baby dyads. All data is presented as median with 25–75^th^ interquartile range.

^a^Two women received multiple antibiotics at delivery including: metronidazole, cephazolin and benzylpenicillin.

**Table 2:** Characteristics of mothers and neonates according to high similarity and lower similarity to maternal oral microbiota composition.

Clinical characteristics	High similarity (n = 19)	Low similarity (n = 17)	P-value
Maternal Age (years)	34.0 (33.0–36.0)	35.5 (30.8–38.8)	0.22
Maternal BMI (kg/m^2^)	30.7 (26.8–32.6)	30.9 (27.8–35.6)	0.35
Mode of delivery^a^			
Vaginal	11/19 (57.9%)	9/17 (52.9%)	1.00
Caesarean	8/19 (42.1%)	8/17 (47.1%)	
Gestational weight gain (kg)	9.1 (7.3–13.5)	7.7 (6.1–14.2)	0.59
Antibiotics during delivery	9/19 (47.3%)	14/17 (82.4%)	**0.04**
Gestational age at delivery	39.6 (38.4–40.7)	39.9 (38.4–40.7)	0.94
Infant birth weight (g)	3686 (3438–3965)	3640 (3158–4086)	0.51
Infant fat composition (g)	10.6 (9.2–16.1)	13.3 (10.1–16.3)	0.48
Gender^a^			
Female	6/19 (31.6%)	7/17 (41.2%)	0.73
Male	13/19 (68.4%)	10/17 (58.8%)	
Expression of *Vim*-1 gene	0/19 (0%)	6/17 (35.3%)	**0.01**

Clinical characteristics of mother-baby dyads. All data is presented as median with 25–75^th^ interquartile range. Statistical significance between group 1 and Group 2 is bolded.

^a^Fisher’s exact test was used to determine significance between the two groups.
