# Corrigendum: Antibiotic treatment at delivery shapes the initial oral microbiome in neonates

**DOI:** 10.1038/srep45615

**Published:** 2017-04-10

**Authors:** Luisa F. Gomez-Arango, Helen L. Barrett, H. David. McIntyre, Leonie K. Callaway, Mark Morrison, Marloes Dekker Nitert

Scientific Reports 7: Article number: 4348110.1038/srep43481 published online: 02 27 2017; updated: 04 10 2017.

In the Supplementary Information file originally published with this Article, Supplementary Figures 3, 4, 5, 6, and 7 were incorrectly labelled as Supplementary Figures 1, 2, 3, 4, and 5 respectively. These errors have now been corrected in the Supplementary Information that now accompanies the Article.

