# Supplementary material for: Antibiotic treatment at delivery shapes the initial oral microbiome in neonates
Source: Sci Rep. 2017 Feb 27;7:43481. doi: 10.1038/srep43481 (PMC5378909; doi:10.1038/srep43481)

Antibiotic treatment at delivery shapes the initial oral microbiome in neonates

*Luisa F. Gomez-Arango [1,2], Helen L. Barrett [1,2,3], H. David. McIntyre [1,4], Leonie K. Callaway [1,2,3], Mark Morrison [5], Marloes Dekker Nitert *, [2,6] for the SPRING trial group*

1. School of Medicine, The University of Queensland, Brisbane Australia
2. UQ Centre for Clinical Research, The University of Queensland, Brisbane Australia
3. Obstetric Medicine, Royal Brisbane and Women's Hospital, Brisbane Australia
4. Mater Research, The University of Queensland, Brisbane Australia
5. Diamantina Institute, Faculty of Medicine and Biomedical Sciences, The University of Queensland, Brisbane Australia
6. School of Chemistry and Molecular Biosciences, The University of Queensland, Brisbane Australia

Table S1. Proportion of maternal oral microbiota in each infant's oral microbiota using bacterial source tracking. Proportions with its mean ± s.d for each infant are shown in the table.

	Proportion oral mom	Proportion_SD_oral mom
1	0.9418	0.015302868
2	0.0625	0.004034573
3	0.6154	0.011481386
4	0.2196	0.005460973
5	0	0
6	0.2757	0.00984378
7	0.2478	0.003765339
8	0.9505	0.008181958
9	0.9852	0.007495184
10	0.0173	0.012018966
11	0.9612	0.004049691
12	0.0985	0.003922867
13	0.71	0.010230673
14	0.5824	0.027277178
15	9.00E-04	0.002024846
16	0.9883	0.007087548
17	0.9911	0.005546771
18	0.9563	0.006481598
19	0.5211	0.015821575
20	0.8112	0.010664583
21	0.9898	0.001813529
22	0.9251	0.009073159
23	1	0
24	1	0
25	0.5263	0.010339246
26	1	0
27	0.0808	0.005006662
28	0.0993	0.00388873
29	1	0
30	0.0514	0.009547542
31	0.9927	0.005100109
32	1	0
33	1	0
34	0.9327	0.012490441
35	1	0
36	0.9898	0.004289522

Table S2. List of primer pairs and probe for amplification of antibiotic resistance genes in neonatal DNA samples.

Gene	***Primer Sequences***	***Annealing temperature***
Vim-1	F: 5′ - AGT GGT GAG TAT CCG ACA G -3′<br>R: 5′ - ATG AAA GTG CG TGGA GAC -3′	53°C
Cmy-2	F: 5′ - TTC TCC GGG ACA ACT TGA CG -3′<br>R: 5′ - GCA TCT CCC AGC CTA ATC CC -3′	58.6°C
Oxa-1	F: 5′ - TAT CTA CAG CAG CGC CAG TG -3′<br>R: 5′ - TGC GTT GCA CAC TTT GCT TT -3′	55.7°C
Shv	F: 5′ - ATT TGT CGC TTC TTT ACT CGC -3′<br>R: 5′ - TTT ATG GCG TTA CCT TTG ACC -3′	60°C
Tem	F: 5′ - ATGAGTATTCAACATTTCCGTG -3′<br>R: 5′ - TTACCAATGCTTAATCAGTGAG -3′	55°C
QuantiTect Probe PCR		
Bla_{Vim}	F: 5′ -CGC GGA GAT TGA GAA GCA AA -3′<br>R: 5′ -AGC CGC CCG AAG GAC ATC -3′	60°C
Vim probe	5′ - (HEX) TTG GAC TTC CTG TAA CGC GTG CA (BHQ1) -3′	-

Fig S3. Differences in microbial taxa distribution at phylum level between group 1 and group 2. **S3a**. Microbial taxa distribution at phylum level between group 1 and group 2. **S3b**. Significant different phyla are shown as boxplots (25th and 75th percentile with a line in the median) Pair-wise comparison were done by t-test and annotated as * : $p < 0.05$, ** : $p < 0.01$, *** : $p < 0.001$

3a

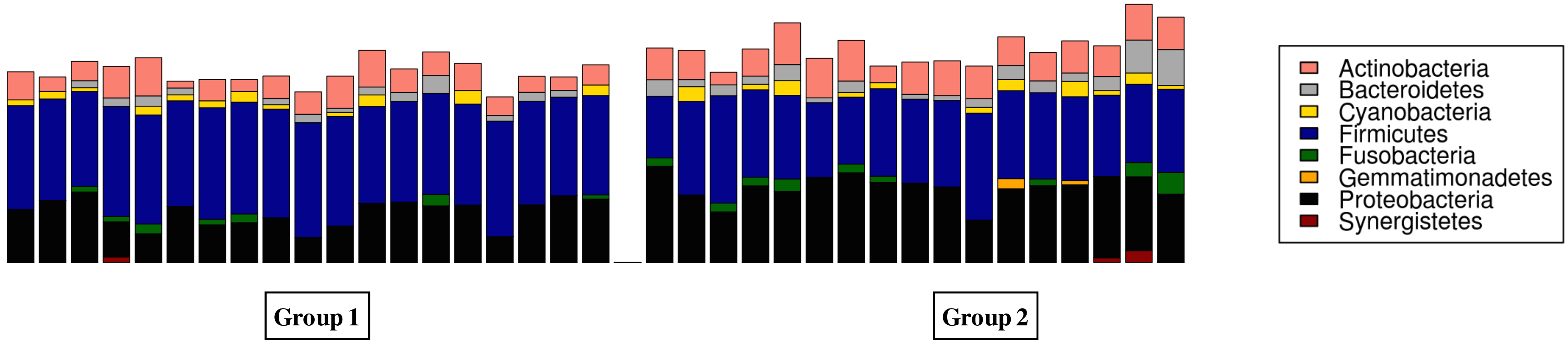

3b

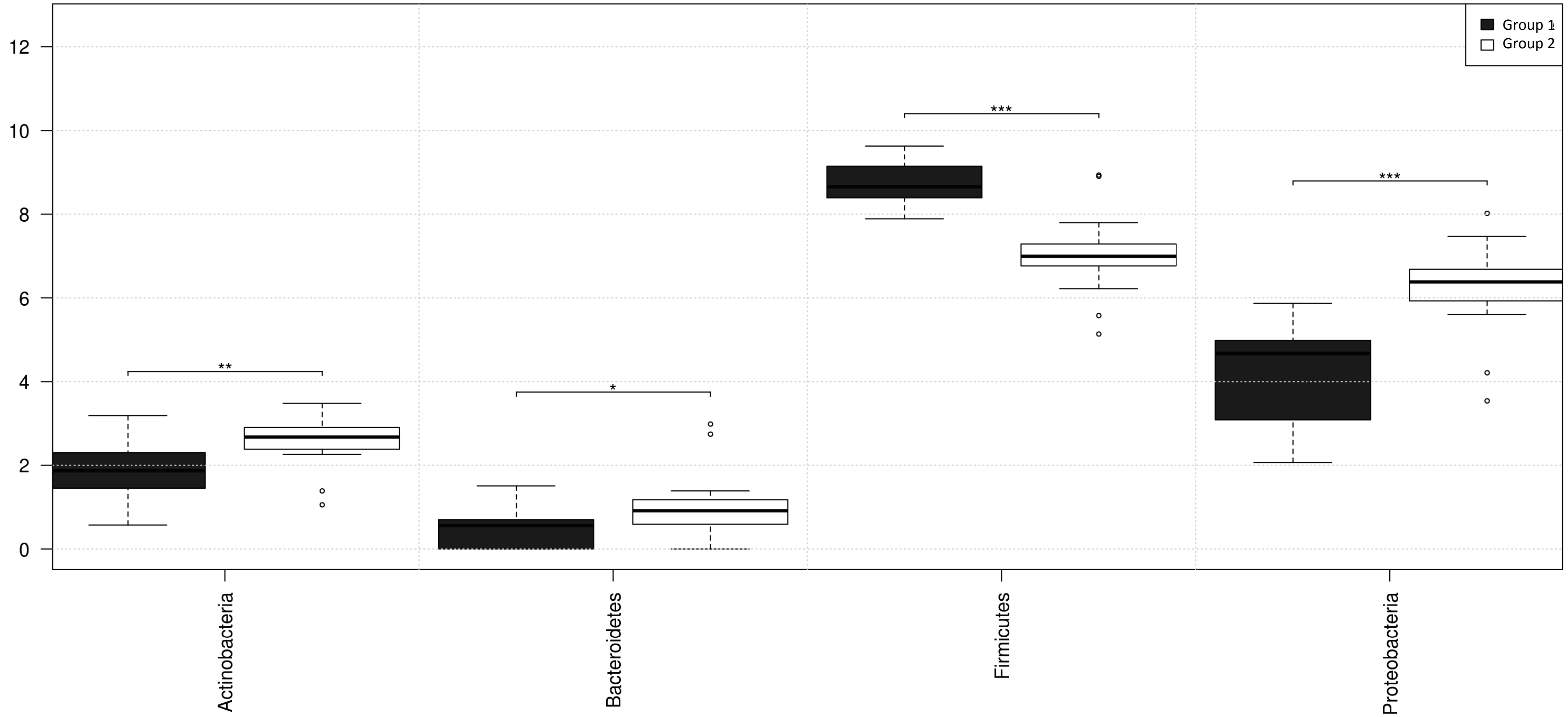

Fig S4: Comparison of infant oral microbiota diversity within group 1 (light grey) and group 2 (black) estimated by Chao1 index (p = 0.0029) and Shannon index (p < 0.0001). Boxplots shows the 25th and 75th percentile with a line at median.

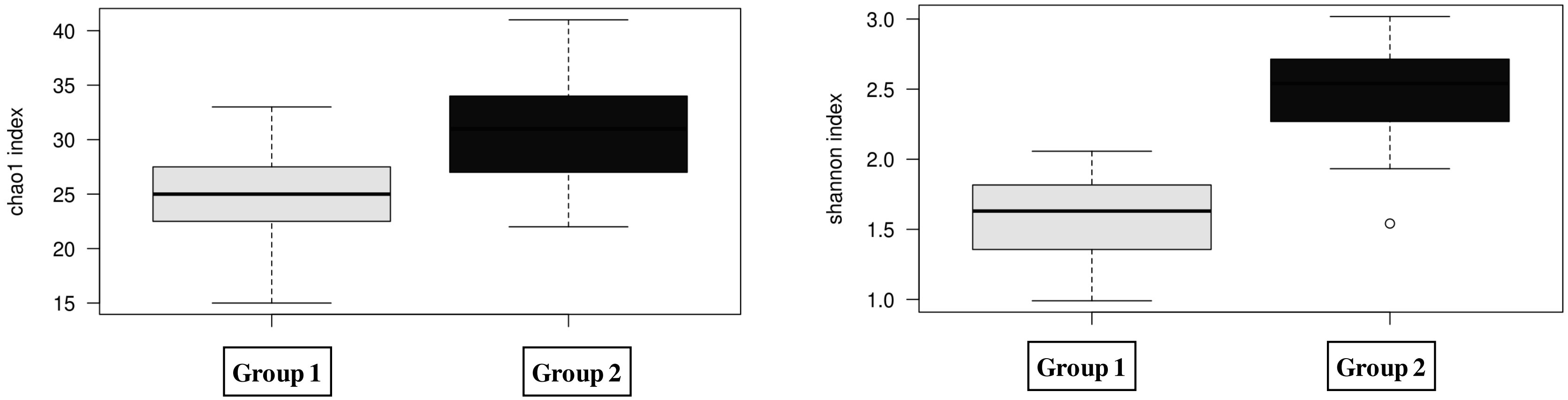

Fig S5. LDA score for differentially abundant taxa in group 1 (red) compared to group 2 (green). Log LDA > 3.0

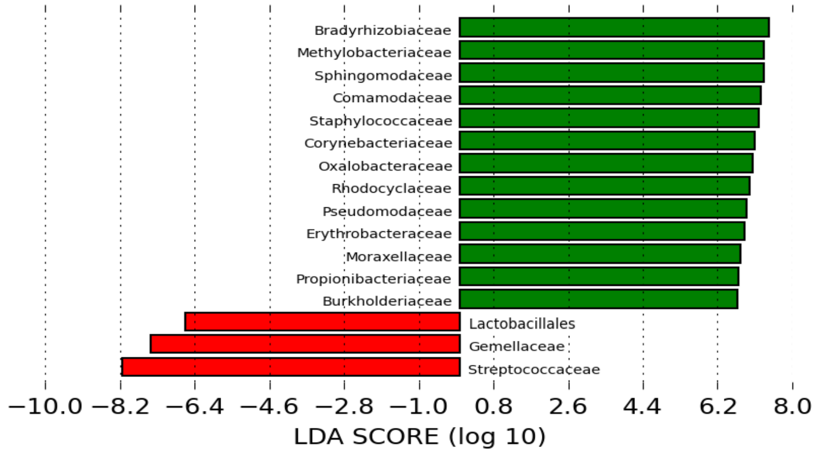

Fig S6. Principal component analysis (PCA) plot at family level for infants born vaginally (red dots) and by caesarean (blue triangles). Non significant clustering was reported for mode of delivery (R = 0.03, p = 0.70)

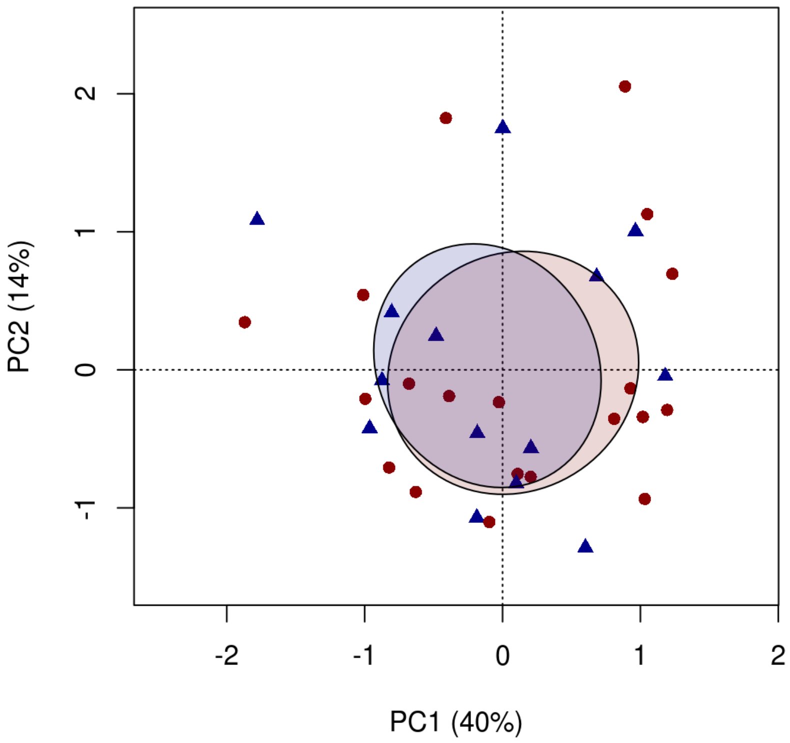

Fig S7. Principal component analysis (PCA) plot at family level based on intrapartum antibiotic used. Benzylpenicillin (blue), Cephazolin (red) and Cocktail (Benzylpenicillin + Cephazolin + Metronidazole) (grey). Non significant clustering was reported for mode of delivery (R = 0.02, p = 0.23).

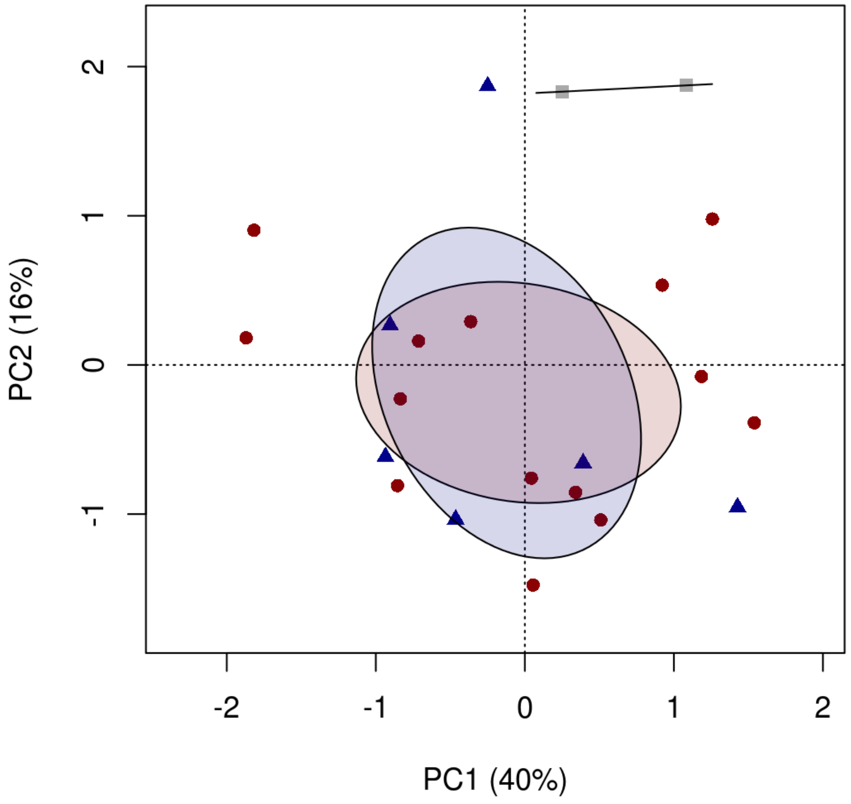

Supplement: Supplementary Information [file srep43481-s1.pdf]
